# Melatonin vs. dexmedetomidine for sleep induction in children before electroencephalography

**DOI:** 10.3389/fped.2024.1362918

**Published:** 2024-04-25

**Authors:** Katja Peganc Nunčič, David Neubauer, Jasna Oražem Mrak, Mirjana Perković Benedik, Urška Mahne, Neli Bizjak, Zvonka Rener Primec, Nataša Šuštar, Tita Butenko, Eva Vrščaj, Damjan Osredkar

**Affiliations:** ^1^ Department of Pediatric Neurology, University Children’s Hospital, University Medical Centre Ljubljana, Ljubljana, Slovenia; ^2^ Faculty of Medicine, Center for Developmental Neuroscience, University of Ljubljana, Ljubljana, Slovenia; ^3^ Department of Pediatric Intensive Care, University Children’s Hospital, University Medical Centre Ljubljana, Ljubljana, Slovenia

**Keywords:** melatonin, dexmedetomidine, electroencephalography, EEG, sleep, sleep induction

## Abstract

**Background and objectives:**

In children requiring electroencephalography (EEG), sleep recording can provide crucial information. As EEG recordings during spontaneous sleep are not always possible, pharmacological sleep-inducing agents are sometimes required. The aim of the study was to evaluate safety and efficacy of melatonin (Mel) and dexmedetomidine (Dex; intranasal and sublingual application) for sleep induction prior to EEG.

**Methods:**

In this prospective randomized study, 156 consecutive patients aged 1–19 years were enrolled and randomized by draw into melatonin group (Mel; *n* = 54; dose: 0.1 mg/kg), dexmedetomidine (Dex) sublingual group (DexL; *n* = 51; dose: 3 mcg/kg) or dexmedetomidine intranasal group (DexN; *n* = 51; dose: 3 mcg/kg). We compared the groups in several parameters regarding efficacy and safety and also carried out a separate analysis for a subgroup of patients with complex behavioral problems.

**Results:**

Sleep was achieved in 93.6% of participants after the first application of the drug and in 99.4% after the application of another if needed. Mel was effective as the first drug in 83.3% and Dex in 99.0% (*p* < 0.001); in the subgroup of patients with complex developmental problems Mel was effective in 73.4% and Dex in 100% (*p* < 0.001). The patients fell asleep faster after intranasal application of Dex than after sublingual application (*p* = 0.006). None of the patients had respiratory depression, bradycardia, desaturation, or hypotension.

**Conclusions:**

Mel and Dex are both safe for sleep induction prior to EEG recording in children. Dex is more effective compared to Mel in inducing sleep, also in the subgroup of children with complex behavioral problems.

**Clinical Trial Registration:**

Dexmedetomidine and Melatonin for Sleep Induction for EEG in Children, NCT04665453.

## Introduction

Electroencephalography (EEG) provides important information regarding brain function in health and disease. Standard 20 channel EEG is an essential tool for timely diagnosis of seizures/epilepsy and evaluation of the treatment effect and can be extremely helpful in other conditions such as acute encephalopathy, infection, or brain lesion (1). Routine EEG recording during wakefulness provides valuable information, but EEG recording during sleep offers important additional and sometimes crucial information about brain function. Sleep generally increases the likelihood of epileptiform activity, especially during the transition between wakefulness and sleep, and vice versa (2, 3). In most epilepsy syndromes the epileptiform discharges are activated during non-rapid eye movements (NREM) sleep (e.g., self-limited epilepsy with centrotemporal spikes, SeLECTS), may occur exclusively during sleep (e.g., electrical status epilepticus during slow-wave sleep, ESES), or after waking from sleep (e.g., juvenile myoclonic epilepsy, West syndrome) are common (4). Recording EEG in spontaneous sleep in children is not always possible, and usually requires a great deal of patience and time (3). Pharmacological sleep inducing agents are therefore often utilized to record EEG in sleep (3, 5), as well as to reduce stress and minimize the presence of movement artefacts on EEG in children, particularly those who cannot cooperate for various reasons, such as complex behavioral problems (2, 3, 5, 6).

Various pharmacological agents can be used for sleep induction prior to EEG recording. Historically, chlorpromazine or chloralhydrate have been used, but have unfavorable safety profiles. Chlorpromazine is linked to extrapyramidal symptoms and must be administered intramuscularly (7), while chloralhydrate is genotoxic (8). These two drugs also interfere with the interpretation of EEG, as they cause slowing of background activity (9, 10). Oral melatonin (N-acetyl-5-methoxytryptamine; Mel) is often utilized for this purpose, as it has a much better safety profile and does not affect EEG (11). It is a hormone physiologically secreted in humans of all ages and is produced by the pineal gland from tryptophan in a circadian pattern (12). It influences the regulation of the hypothalamic-pituitary-adrenal system, regulates circadian rhythms, secretion of other hormones and body temperature (13). When given exogenously, it acts as an analogue of natural melatonin inducing sleep (3, 14). Side effects are extremely rare and occur only at extremely high doses (15). However, melatonin sometimes fails to induce or maintain sleep, especially in patients with complex behavioral problems related to developmental delay, autism spectrum disorder, and intellectual disability (16). Dexmedetomidine (Dex) is a specific agonist for the alfa-2 receptors in the locus coeruleus, that activates endogenous pathways responsible for sleep (17, 18). It stimulates the activation of inhibitory neurons secreting γ-aminobutanoic acid or γ-aminobutyric acid, inducing a state similar to the second stage of natural sleep (17, 18). Dex is commonly used for sedation in intensive care units and for procedural sedation in children (19). It provides sedation without respiratory depression and major effects on the cardiovascular system, has minimal effects on EEG peak frequency and amplitude, does not affect seizures or alter spike-wave activity (20–22). However, Dex has not been extensively used for sleep induction prior to EEG in pediatric patients and it's safety for this purpose has not been clearly established.

This prospective randomized trial aimed to compare the efficacy and safety of Mel and Dex for sleep induction before EEG in children. The utility of both drugs in children with behavioral problems was of particular interest in this study. Two routes of Dex administration were studied (sublingual and intranasal).

## Methods

This prospective randomized study was approved by the National Medical Ethics Committee of Slovenia (0120-597/2019/16) and was registered at **clinicaltrials.gov** (NCT04665453). Informed written consent was obtained from all participants and/or their legal guardians. None of the participants received any compensation for their participation.

### Participants

This study was conducted at the Department of Pediatric Neurology, University Children's Hospital, University Medical Centre Ljubljana, Ljubljana, Slovenia, between September 2020 and September 2022. The cohort consisted of a consecutive series of patients aged 1–19 years who were referred to the department for EEG recording during wakefulness and sleep by a pediatric neurologist. All the children who fulfilled the inclusion criteria were invited to participate in the study. Exclusion criteria were inability to follow the study protocol and/or use of the following medications: digoxin, beta-receptor blockers, or calcium channel blockers. None of the patients received either drug in the last one week prior to EEG, if at all. The patients were not instructed to be fasted before EEG recording.

A subgroup of patients with complex behavioral problems was identified, including patients with intellectual disability, pervasive developmental disorder, and/or developmental delay.

### Randomization

All enrolled participants were randomized by envelope drawing into one of three treatment groups: oral melatonin group (Mel; dose 0.1 mg/kg), sublingual dexmedetomidine group (DexL, dose 3 mcg/kg), or intranasal dexmedetomidine group (DexN, dose 3 mcg/kg). The patient, guardians, and researchers were not blinded to treatment selection. We aimed to enroll at least 50 participants per group, for a total of at least 150 participants.

### EEG recording and sleep induction

Patient preparation and recording took place at the department's EEG laboratory in a quiet room with low-light levels. All recordings were performed in the same room, under the same conditions. Younger children were usually in their parents' arms during preparation and recording, while older children were usually laying alone on the bed (and sometimes accompanied by a parent). Before the selected drug was administered, 3 ECG electrodes, a cuff for pressure measurement, and a pulse oximeter were attached, as well as an EEG cap with international system 10–20 electrodes and a breathing electrode. The drugs were administered by a nurse. The time of administration, the time at which the participant reached the 2nd NREM sleep stage, and waking up were recorded, as well as any problems related to the recording. In case the participant had not reached at least the first sleep stage 30 min after the administration of the first drug, the second drug was chosen: the participants who received Dex as the first drug received Mel as the second drug, and those who received Mel as the first drug were given Dex (route of administration was again randomly chosen). The pediatric neurologist who evaluated all the EEG recordings (DN) had several decades of experience in pediatric EEG and sleep recording and was blinded to the treatment the child received prior to EEG recording. Besides parameters related to the transition from wakefulness to sleep, no other EEG qualities were evaluated for the purpose of this study.

### Vital signs

The safety of the two compounds was checked based on measured vital signs (heart rate, blood pressure, respiratory rate, and blood oxygen level). Heart rate, respiratory rate, and SpO_2_ (oxygen saturation as measured by pulse oximetry) were recorded every 10 min (min) until the end of the EEG recording and 120 min after administration with a Philips IntelliVue MP50 monitor. Blood pressure was measured at the time of drug administration, immediately after the EEG recording ended, and after 120 min, to avoid waking the subjects during measurements. Reference data from the Pediatric Advanced Life Support guidelines were used as the benchmark. (23).

### Statistics

The statistical program “Statistical Package for the Social Sciences” (SPSS) 26.0 was used for statistical analysis. Prism 9, version 9.5.1, was used for the figures. First, descriptive statistics were performed using the Shapiro-Wilk test, and the results were compared and analyzed between groups using the *t*-test, chi-squared test, non-parametric tests, or ANOVA (with appropriate post hoc tests). Statistical significance was set at *p* < 0.05.

## Results

### Study population

Informed consent was obtained from 166 patients in period between September 2020 and September 2022, but 10 were excluded because of protocol deviation (poor cooperation). The median age of the children was 5.5 years (range 1.0–18.9), of which 52 (33.3%) were female and 104 (66.7%) were male. The enrolled participants were randomized as follows: 54 patients in the Mel group, 51 in the DexL group, and 51 in the DexN group. The list of diagnoses for which the children were referred for EEG is presented in Table 1 (multiple diagnoses were possible in a single patient). Other diagnoses include genetic mutations, chromosomopathies, tuberous sclerosis, polymicrogyria, cerebral infarction, and developmental speech disorders. In the subgroup of children with complex behavioral problems, 65 participants were included, of whom 46 were male and 19 were female.

**Table 1:** Diagnoses of patients referred for EEG in wakefulness and sleep.

	Epilepsy (77)	First epileptic seizure (38)	Febrile convulsions (6)	Develop -mental delay (37)	Intelectual disability (22)	Pervasive develop—mental disorder (26)	Cerebral palsy (16)	Other (58)
Mel	27	15	4	10	4	5	3	17
DexL	23	13	0	16	9	9	9	18
DexN	27	10	2	11	9	12	4	23

Mel, melatonin; DexL, dexmedetomidine administered sublingually; DexN, dexmedetomidine administered intranasally.

### Sleep induction

**Figure 1:**
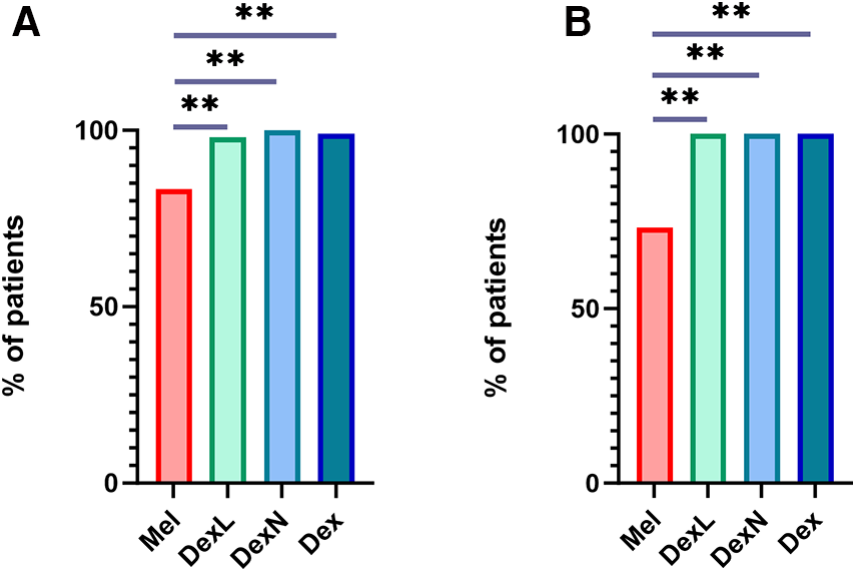
Successful sleep induction after the application of the first drug. (**A**) Successful sleep induction in all participants. (**B**) Successful sleep induction in subjects with complex behavioural issues. Mel, melatonin; DexL, dexmedetomidine administered sublingually; DexN, dexmedetomidine administered intranasally; Dex, dexmedetomidine combined; **p ≤ 0.001.

Sleep was achieved in 93.6% of participants after the application of the first drug and in 99.4% of participants after the application of the second drug if needed. Only one subject (0.6%) did not fall asleep after receiving both drugs. After receiving Mel, 45/54 subjects fell asleep (83.3%). After receiving DexN, all subjects fell asleep, whereas after receiving DexL, one subject did not fall asleep, resulting in a total of 101/102 (99.0%) of participants falling asleep after Dex. In terms of efficacy, DexL and DexN did not differ significantly (*p* = 0.32). Dex was more effective in inducing sleep compared to Mel (*p* < 0.001). The efficacy of sleep induction after application of the first drug is shown in Figure 1A.

Mel was used as a second drug in only one case, whereupon the subject fell asleep. After unsuccessful sleep induction with Mel, DexL was used in two cases as the second drug (both subjects fell asleep), and DexN was used in seven cases (6/7 subjects fell asleep).

Analysis of data for a subgroup of children with complex behavioral problems showed that Mel was successful as a first-use medication in 11/15 (73.4%) children, while DexL and DexN were successful in 100% (Figure 1B). Thus, even in this subpopulation, Dex proved to be a more effective drug for inducing sleep than Mel (*p* < 0.001).

**Figure 2:**
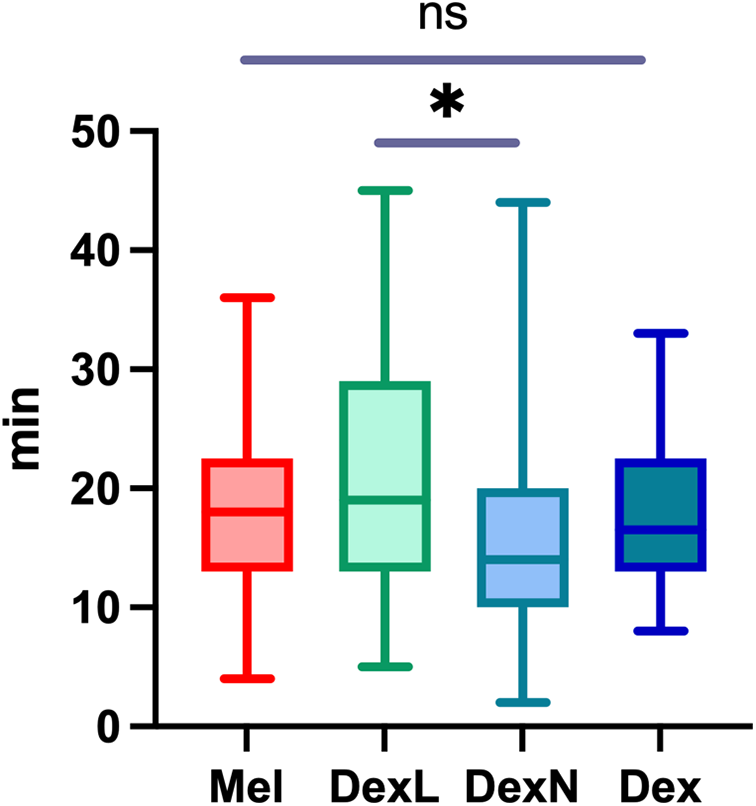
The time from the application of the first drug to reaching NREM2 sleep stage. Mel, melatonin; DexL, dexmedetomidine administered sublingually; DexN, dexmedetomidine administered intranasally; Dex, dexmedetomidine combined; NREM2, non rapid eye movement sleep phase 2; *p < 0.05; ns, not significant.

The average time to reach the NREM sleep phase 2 (NREM2) was 18.0 min after the first application of the drug and 15.9 min after the second application, if required. Considering only children, who fell asleep after the first drug, subjects fell asleep after Mel on average after 17.3 min (range, 4–36 min; SD 7.6 min), after DexL in 20.6 min (range, 5–45 min; SD 9.6 min), and after DexN in 15.7 min (range, 2–44 min; SD 7.9 min). The differences between the groups are shown in Figure 2. A significant difference was only observed between the groups that received Dex via different routes (*p* = 0.006), whereas the differences compared to Mel were not significant (*p* = 0.969). In the subgroup of patients with complex behavioral problems, there were no differences in the time taken to reach NREM2 between the groups. We did not find a significant influence of age on the time to sleep in all patients combined or in subgroups of patients receiving a particular drug. Sleep deprivation of various extent was suggested for 18/156 (11.5%) of patients; sleep deprivation did not significantly affect time to sleep in our cohort.

### Safety/vital functions

None of the monitored values (heart rate, respiratory rate, blood oxygen level, and blood pressure) were outside the normal range, and no respiratory depression, bradycardia, hypotension, or desaturation was observed in any of the subjects. Some of the physiological parameters at the first measurement(s) were probably affected by the fact that many children were restless or even frightened at the beginning of the examination because of the procedures in the unfamiliar environment and being surrounded by unfamiliar people (a common observation even outside the study).

**Figure 3:**
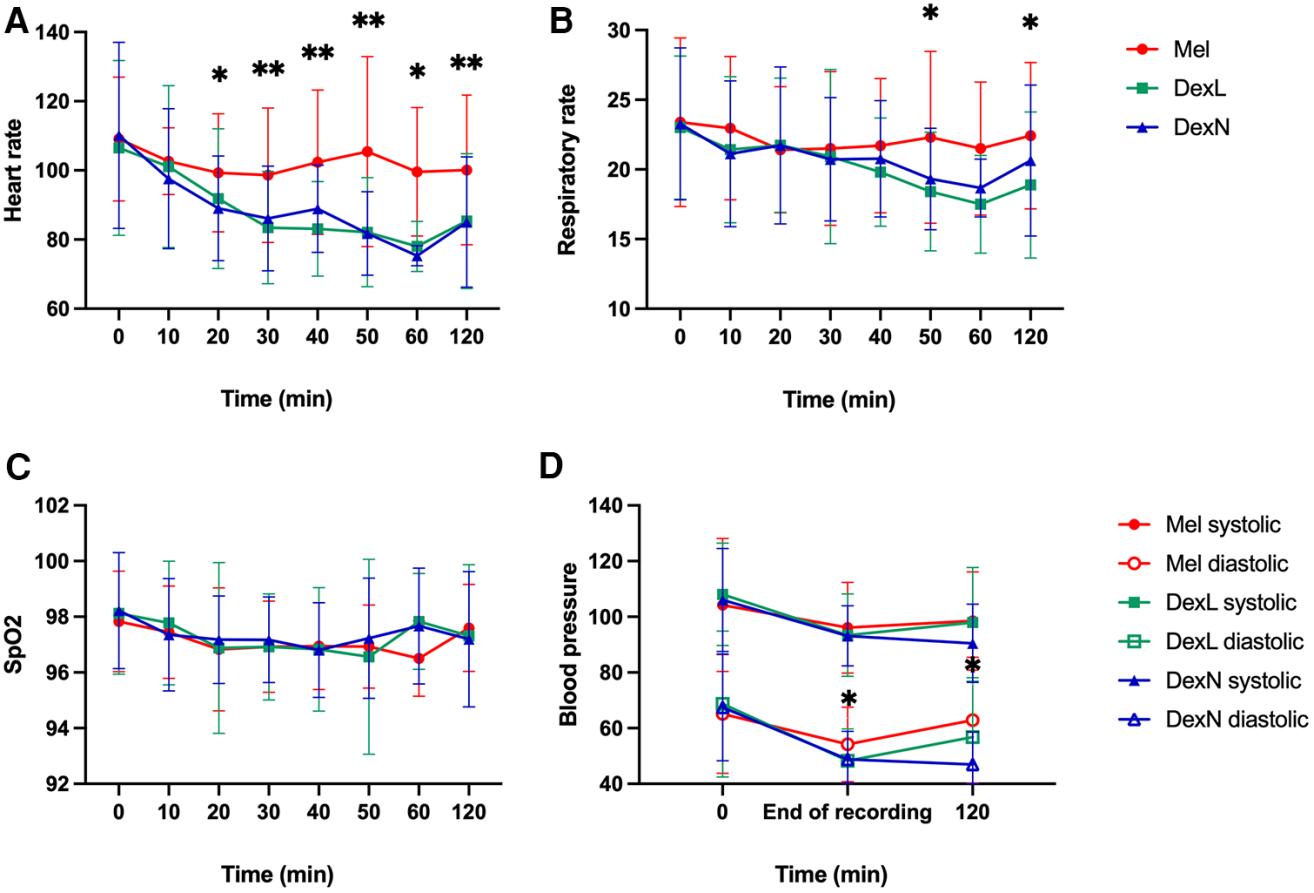
Change of vital functions in time. (**A**) Change of heart rate from time of drug administration until 120 min after. (**B**) Change of respiratory rate from time of drug administration until 120 min after. (**C**) Change of SpO_2_ from time of drug administration until 120 min after. (**D**) Change of blood pressure from time of drug administration until 120 min after. Mel, melatonin; DexL, dexmedetomidine administered sublingually; DexN, dexmedetomidine administered intranasally; Dex, dexmedetomidine combined; SpO_2_—oxygen saturation as measured with pulse oximetry; *p < 0.05; **p ≤ 0.001.

For the heart rate, at time points 0 and 10 min after administration there were no significant differences between the groups, while later on there were significant differences between Mel and Dex groups: patients receiving Dex by any route of administration had lower heart frequencies (after 10 and 60 min, *p* = 0.003; after 20, 30, 40, 50, 60, 120 min and at end, *p* < 0.001; Figure 3A). There were no significant differences in respiratory rate between the groups at any time point, except for measurements at 50 min (*p* = 0.025) and 120 min (*p* = 0.012) after administration, where patients receiving Dex by any route had lower respiratory rates (Figure 3B). The mean blood oxygen level was always above 94% in all groups, and no significant differences were found between the groups (Figure 3C). There were no significant differences in blood pressure between the groups, except for diastolic values at the end of the recording, where values after receiving DexL and DexN were lower compared to Mel (*p* = 0.018 and *p* = 0.025, respectively); after 120 min there were lower values of diastolic blood pressure after DexN compared to Mel (*p* = 0.001) and when compared to DexL (*p* = 0.021) (Figure 3D). More subjects were still asleep 120 min after sleep induction if they received Dex than Mel (numbers not shown).

## Discussion

The results of the present study suggest, that both, Mel and Dex are safe and effective drugs for sleep induction in children undergoing EEG, but there are differences in efficacy: in general, Dex was more effective than Mel in inducing sleep (i.e., more children fell asleep), although the time for sleep induction was not different between Mel and Dex. This was also true for a subpopulation of patients with complex behavioral problems. In the case of failure to fall asleep after receiving the first drug, it is useful to add the other (if the child does not fall asleep after Mel, it makes sense to give Dex and vice versa).

If we compared the efficacy after the application of first drug, we see that Dex was more efficient than Mel in sleep induction, as 99.0% of patients fell asleep after Dex while only 83.3% fell asleep after Mel (*p* < 0.001). To the best of our knowledge, this is the first study to compare the efficacy of these two drugs in sleep induction side-by-side prior to EEG. Our results regarding the efficacy of Mel for sleep induction prior to EEG were similar to the results of other studies, which ranged between 70% and 83% (24–28). In several studies, Dex has also been shown to be successful in inducing sleep prior to EEG in children (24–28). In a study using the same dose as in our study, sleep was successfully achieved in 92.9% of subjects when Dex was administered orally (29), and in 90.4% and 87% of subjects when it was administered intranasally (30, 31), which was lower than that in our study. Our study adds that if one drug is not effective, adding the other appears to be a successful strategy for inducing sleep in most patients, and this method appears to be safe.

When a subgroup of children with complex behavioral problems was analyzed separately, Dex was again more effective than Mel at inducing sleep. Yuena et al. came to a similar conclusion in a study comparing Mel and chloral hydrate as sedating agents in children undergoing EEG. In their study, Mel was less effective than chloral hydrate in sleep induction (83% vs. 87%, respectively), and in the subgroup of children with developmental delay, cerebral palsy, and intellectual disability the percentage of failure after Mel was higher (32). When assessing the time lag between drug administration and sleep induction, there were no differences between Mel and Dex, although within the Dex group our data suggest that intranasal application could have a faster effect. Cimen et al. came to a similar conclusion: intranasal application was more effective than buccal administration because drugs might be swallowed by uncooperative children before there is sufficient time for absorption (33). This could be of importance in children with behavioral problems to lower the burden of EEG recordings in this subpopulation.

Both Mel and Dex were safe to use and none of the participants experienced any significant side effects. We have observed lower diastolic blood pressures in children receiving Dex, but this is possibly related to the fact, that more participants were still asleep after 120 min post sleep induction if they received Dex, compared to Mel. However, some studies have shown that Dex has an effect on the cardiovascular system and could be responsible for lowering the heart rate and blood pressure, while the effect on the respiratory rate and saturation is less common (21, 24–26, 34–39). Nevertheless, our findings are in line with several studies which have demonstrated, that Dex is safe to use in children undergoing EEG (40–42). However, awareness of the possible adverse reactions is essential to prevent potential complications. Mel, on the other hand, minimally affected vital signs in our study, nor did we find any studies showing that it significantly affected any of the vital signs, therefore it appears to also be a safe drug for sleep induction in children (14, 32, 43–45).

This study had some limitations. Although the patients were randomized to treatment groups, the patients/parents and healthcare providers (except the pediatric neurologist who evaluated EEG recordings) were not blinded to the treatment in a particular child, which could be a source of bias. Children were recorded in an unfamiliar environment, and some experienced anxiety, fear, and restlessness, which could affect the measurement of physiological parameters. Although studies have shown that the effect of both drugs on EEG recording is minimal (i.e., background activity) (11), this was not examined in the present study while it would be interesting to further analyze this. Similarly, a more detailed analysis of sleep stages following sleep induction was not the focus of the present study, although it could provide further information.

## Conclusion

Mel and Dex are safe and efficient for sleep induction prior to EEG recording in children undergoing EEG. Dex has been shown to be more effective than Mel in inducing sleep, particularly in children with complex behavioral problems, but also has a greater effect on vital signs, although none of the parameters were out of the physiological range. The most effective way to achieve induced sleep in children prior to EEG recording is intranasal application of Dex.

## Data Availability

The raw data supporting the conclusions of this article will be made available by the authors, without undue reservation.
