# Supplementary figures and images for: Melatonin vs. dexmedetomidine for sleep induction in children before electroencephalography
Source: Front Pediatr. 2024 Apr 25;12:1362918. doi: 10.3389/fped.2024.1362918 (PMC11079286; doi:10.3389/fped.2024.1362918)

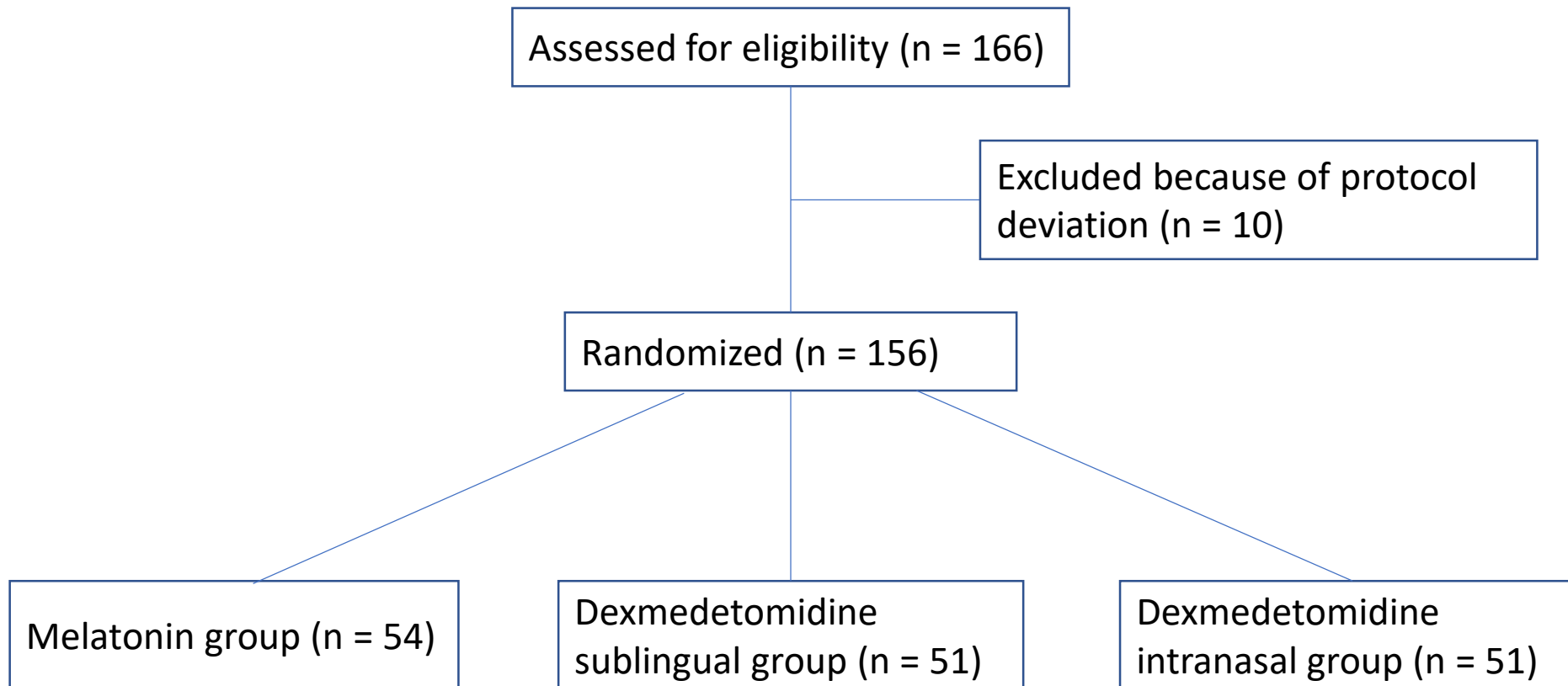

Supplement: Supplementary file 1 [file Datasheet1.pdf]
